# Lipoxin A_4_ suppresses angiotensin II type 1 receptor autoantibody in preeclampsia via modulating caspase-1

**DOI:** 10.1038/s41419-020-2281-y

**Published:** 2020-01-30

**Authors:** Haojing Liu, Fangxiong Cheng, Qiang Xu, Wei Huang, Sumei Wang, Rui Sun, Duyun Ye, Dongxin Zhang

**Affiliations:** 1 Department of Rheumatology and Immunology, Wuhan Fourth Hospital, Puai Hospital, Tongji Medical College, Huazhong University of Science and Technology, Wuhan, 430033 PR China; 2 Department of Clinical Laboratory, Wuhan Fourth Hospital, Puai Hospital, Tongji Medical College, Huazhong University of Science and Technology, Wuhan, 430033 PR China; 3 Department of Pathophysiology, Tongji Medical College, Huazhong University of Science and Technology, Wuhan, 430030 PR China; 4 Department of Oncology, Wuhan Fourth Hospital, Puai Hospital, Tongji Medical College, Huazhong University of Science and Technology, Wuhan, 430033 PR China

**Keywords:** Cell death and immune response, Reproductive disorders, Inflammasome

## Abstract

Preeclampsia (PE) remains a leading cause of maternal and neonatal morbidity and mortality. Numerous studies have shown that women with PE develop autoantibody, termed angiotensin II type 1 receptor autoantibody (AT1-AA), and key features of the disease result from it. Emerging evidence has indicated that inflammatory cell necrosis, such as pyroptosis, could lead to autoantigen exposure and stimulate autoantibody production. Caspase-1, the central enzyme of inflammasome and key target of pyroptosis, may play roles in AT1R exposure and AT1-AA production. Exploring endogenous regulator that could inhibit AT1-AA production by targeting pyroptosis will be essential for treating PE. Lipoxin A_4_ (LXA_4_), endogenous dual anti-inflammatory and proresolving lipid mediator, may inhibit AT1-AA production via modulating caspase-1. Thus, we explore whether caspase-1 is essential for AT1-AA production and LXA_4_ inhibits AT1-AA via modulating caspase-1. PE patients and mice developed AT1-AA associated with caspase-1 activation. Caspase-1 deletion leaded to AT1-AA decrease in PE mice. Consistent with these findings, we confirmed caspase-1 activation, trophoblast pyroptosis and AT1R exposure in PE mice and trophoblast model, while caspase-1 deficiency showed decreased trophoblast pyroptosis and AT1R exposure in vitro and in vivo. Interestingly, LXA_4_ could suppress AT1-AA production via regulating caspase-1 as well as enhancing phagocytosis of dead trophoblasts by macrophages. These results suggest that caspase-1 promotes AT1-AA production via inducing trophoblast pyroptosis and AT1R exposure, while LXA_4_ suppresses AT1-AA production via modulating caspase-1, supporting caspase-1 serving as a therapeutic target for attenuating AT1-AA and LXA_4_ protecting patients from AT1-AA and PE.

## Introduction

Preeclampsia (PE), characterized by maternal hypertension, proteinuria and other systemic disorders occurring after 20 weeks of gestation, remains a leading cause of maternal and neonatal morbidity and mortality^1–4^. Although the pathogenesis of PE remains largely unknown, immune mechanisms and renin-angiotensin system are implicated in PE^5–9^. These two concepts were united in a previous report showed that serum from PE women contain autoantibodies that bind and activate angiotensin II type 1 receptor (AT1R)^10^. Increasing studies from us and others^7–13^ have shown that PE is a pregnancy-induced autoimmune disease in which key features of the disease result from the autoantibody, termed angiotensin II type 1 receptor autoantibody (AT1-AA). However, previous works have been restricted to confirmation of its pathophysiological relevance to PE and have been unable to specifically clarify the mechanisms for determining AT1-AA production.

Dying cells are rapidly engulfed by phagocytes, a process that prevents inflammation or autoimmune response against intracellular antigens^14,15^. Cell death occurs in healthy individuals during homeostasis yet autoimmunity doesn’t develop, at least in part, because of rapid clearance of dying cells^15,16^. Our previous results together with other findings suggest that accelerated cell death combined with clearance deficiency lead to the accumulation and externalization of autoantigens and autoantibody production^17,18^. A previous report showed that trophoblasts apoptosis is enhanced in PE^19^, and another report indicated that the nature of trophoblast death changes from being apoptosis-like to being more necrotic^20^. Pyroptosis, also termed inflammatory necrosis, is a recently identified caspase-1 dependent cell death, and its induction requires two distinct stimuli, exogenous PAMPs (pathogen-associated molecular patterns) and endogenous DAMPs (damage-associated molecular patterns)^21–23^. In responses to PAMPs and DAMPs, Nod-like receptor proteins (NLRPs) are recruited for the formation of inflammasome, in which procaspase-1 is converted to active caspase-1^21–23^. Evidence has shown that cell death, such as pyroptosis, could stimulate autoantibodies production^18^. Of importance, Kahlenberg et al. found that caspase-1 is involved in autoantibodies production in SLE^24^. Thus, caspase-1 may represent a key target in inducing the exposure of trophoblast autoantigen AT1R and AT1-AA production in PE.

Exploring endogenous regulator that could inhibit AT1-AA production by targeting caspase-1 will be essential for treating PE. Lipoxin A_4_ (LXA_4_), endogenous anti-inflammatory and proresolving lipid mediator derived from arachidonic acid enzymatically by lipoxygenases (LOXs) involving the dual lipoxygenation of arachidonic acid via either ALOX15/ALOX5 or ALOX5/ALOX12, function as “stop signal” in inflammation^25^. Together with our previous studies, increasing findings suggest that LXA_4_ plays vital roles in inflammation termination and homeostasis restoration of reproductive system^26–29^. We found previously that human PE is associated with a deficiency of LXA_4_ that could alleviate PE-related symptoms in rat model^28,29^. Interestingly, a previous report indicated that ALOX15-deficient animals displayed a break of self-tolerance and, with increasing age, developed autoimmune disease^30^. Moreover, loss of ALOX15 activity resulted in an aberrant phagocytosis of apoptotic cells (ACs) by inflammatory monocytes and subsequent presentation of AC-derived autoantigens^30^. ALOX15 can contribute to the generation of LXA_4_, which has been identified as an important factor initiating inflammation resolution and, in turn, is implicated in the removal of ACs^30^. Hence, LXA_4_ may serve as a key modulator for inhibiting AT1-AA production via regulating caspase-1.

Although the adverse effects of AT1-AA on pregnancy have been extensively investigated, the mechanisms for determining AT1-AA production in PE remain largely unclear. Given the importance of caspase-1 in mediating pyroptosis and autoantigen exposure, the purpose of the present study was to investigate whether caspase-1 is essential for AT1R exposure and AT1-AA production and whether LXA_4_ could inhibit AT1-AA production via modulating caspase-1.

## Results

### AT1-AA expression is associated with caspase-1 activation in human PE

**Fig. 1:**
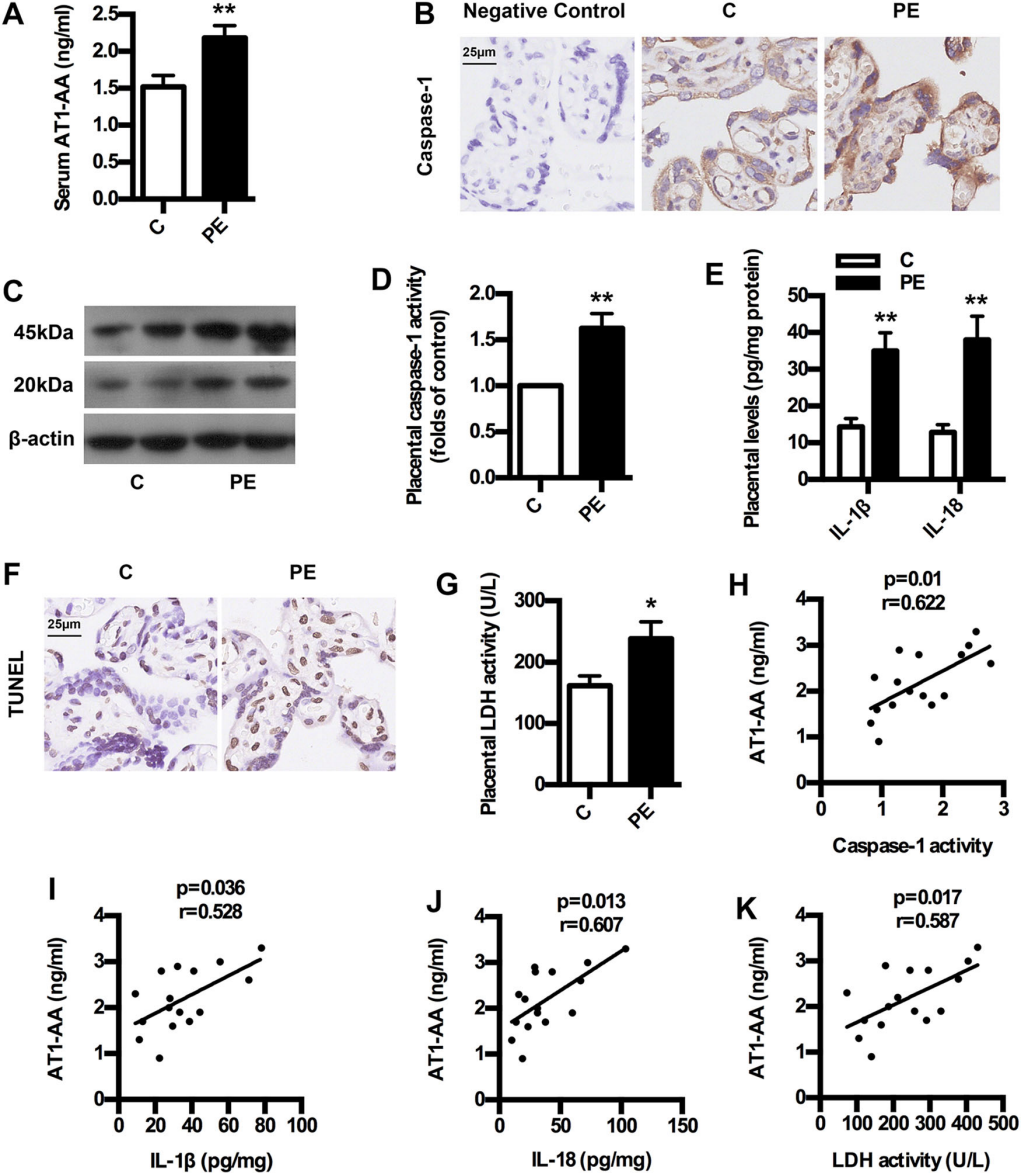
AT1-AA expression, caspase-1 activation and trophoblast pyroptosis in PE patients. **a** Comparison of serum AT1-AA levels between PE patients and healthy controls. **b**, **c** Comparison of placental caspase-1 expressions between PE patients and healthy controls. IHC (**b**) and WB (**c**) analysis of caspase-1 in placenta were shown. Bar = 25 μm. **d** Comparison of placental caspase-1 activity between PE patients and healthy controls. **e** Comparison of placental IL-1β and IL-18 levels between PE patients and healthy controls. IL-1β and IL-18 were detected by ELISA. **f** Comparison of trophoblast death between PE patients and healthy controls. TUNEL analysis in placenta was shown. Bar = 25 μm. **g** Comparison of placental LDH activity between PE patients and healthy controls. **h**–**k** The correlation between AT1-AA and caspase-1 activity, IL-1β, IL-18 and LDH activity in PE women and normal pregnancies. Results are expressed as means ± SEM (*n* = 16 in each group). *p < 0.05 and **p < 0.01 versus control group, two-tailed Student’s *t* test.

To confirm whether AT1-AA expression is correlated with caspase-1 activation in human PE, we determined the levels of AT1-AA and caspase-1 activation and trophoblast pyroptosis in both normal pregnancy and PE women. As shown in Fig. 1a, serum AT1-AA levels in PE showed significant increase compared with controls. Placental caspase-1 expressions upregulated significantly in PE women than normal pregnancies (Fig. 1b, c). In line with this, placental caspase-1 activity was higher in PE women than normal pregnancies (Fig. 1d). Furthermore, placental levels of IL-1β and IL-18 in PE women increased significantly compared to controls (Fig. 1e). Meanwhile, trophoblast death in PE was exacerbated compared to control (Fig. 1f, g). Importantly, there were significant correlations between AT1-AA and caspase-1 activity, IL-1β, IL-18 and LDH activity in PE (Fig. 1h–k). These clinical data suggest that AT1-AA expression is associated with caspase-1-mediated pyroptosis in human PE, implicating caspase-1 activation in AT1-AA production.

### Caspase-1 activation, pyroptosis and AT1R exposure in PE mice and trophoblast model

**Fig. 2:**
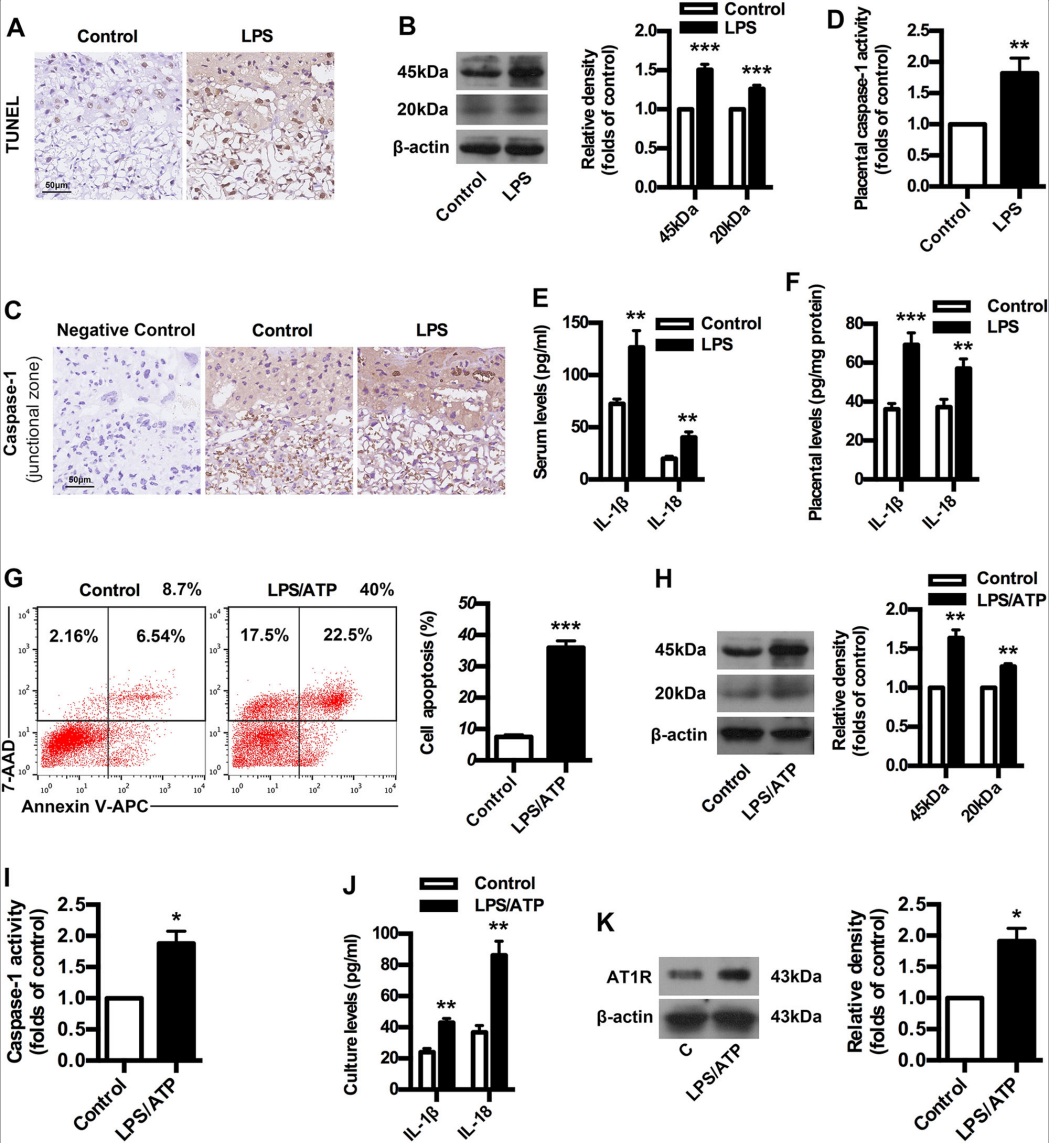
Caspase-1 activation, pyroptosis and AT1R exposure in PE mice and trophoblast model. **a** Comparison of trophoblast death between control and PE mice. TUNEL analysis in placenta was shown. Bar = 50 μm. **b**, **c** Comparison of placental caspase-1 expression between control and PE mice. WB (**b**) and IHC (**c**) analysis of caspase-1 in placenta were shown. The histogram represents means ± SEM of the densitometric scans for protein bands (*n* = 7 mice in each group), normalized by comparison with β-actin and expressed as a percentage of Control. Bar = 50 μm. **d** Comparison of placental caspase-1 activity between control and PE mice. Comparison of IL-1β and IL-18 levels in serum (**e**) and placenta (**f**) between control and PE mice. IL-1β and IL-18 were detected by ELISA. Results are expressed as means ± SEM (*n* = 7 mice in each group). **p < 0.01 and ***p < 0.001 versus control group, two-tailed Student’s *t* test. **g** Effect of LPS/ATP on trophoblast apoptosis. Human first-trimester trophoblast cell line HTR-8/SVneo was treated with LPS and ATP. Cell apoptosis was detected by using flow cytometry. **h** Effect of LPS/ATP on trophoblast caspase-1 expression. Caspase-1 was detected by WB. **i** Effect of LPS/ATP on trophoblast caspase-1 activity. **j** Effect of LPS/ATP on trophoblast IL-1β and IL-18 levels. IL-1β and IL-18 were detected by ELISA. **k** Effect of LPS/ATP on trophoblast AT1R exposure. AT1R was detected by WB. Results are expressed as means ± SEM from three independent experiments. *P < 0.05, **P < 0.01 and ***P < 0.001 versus control group, two-tailed Student’s *t* test.

To validate above clinical data in animal models of PE, an ultra-low-dose LPS-induced PE model was constructed in mice. LPS-treated mice developed PE-related symptoms, including hypertension, proteinuria, fetal intrauterine growth restriction, placental oxidative stress and structural abnormalities in kidney characterized by glomerular endotheliosis (Supplementary Fig. 1a–e). Meanwhile, LPS-treated mice showed placental inflammatory activation and imbalanced angiogenesis, including overexpressed p-P38 and NF-κB as well as increased antiangiogenic factors sFlt-1, sENG and ET-1 (Supplementary Fig. 1f–h). These results suggest the successful construction of PE model in mice. Next, we determined whether caspase-1 activation is involved in AT1-AA production by employing the mice model and trophoblast model. As shown in Fig. 2a, LPS-treated mice had enhanced trophoblast death compared to control. WB and IHC results showed that placental caspase-1 expressions upregulated significantly in PE mice (Fig. 2b, c). In line with this, placental caspase-1 activity was also stimulated in PE mice (Fig. 2d). The levels of IL-1β and IL-18 in both serum and placenta of PE mice increased significantly compared with controls (Fig. 2e, f). Intriguingly, the proportions of late apoptotic/necrotic trophoblast cells were significantly increased after LPS pretreatment and ATP exposure (Fig. 2g). LPS/ATP exposure upregulated trophoblast caspase-1 expressions (Fig. 2h). In line with this, LPS/ATP exposure stimulated trophoblast caspase-1 activity (Fig. 2i). Meanwhile, LPS/ATP exposure increased the levels of IL-1β and IL-18 in cell culture (Fig. 2j). Importantly, the expressions of AT1-AA specific autoantigen AT1R were enhanced by LPS/ATP exposure (Fig. 2k). These results suggest caspase-1 activation, trophoblast pyroptosis and AT1R exposure in both mice model and trophoblast model, indicating the possible involvement of caspase-1 in AT1R exposure and AT1-AA production.

### Increased AT1-AA production in PE mice

**Fig. 3:**
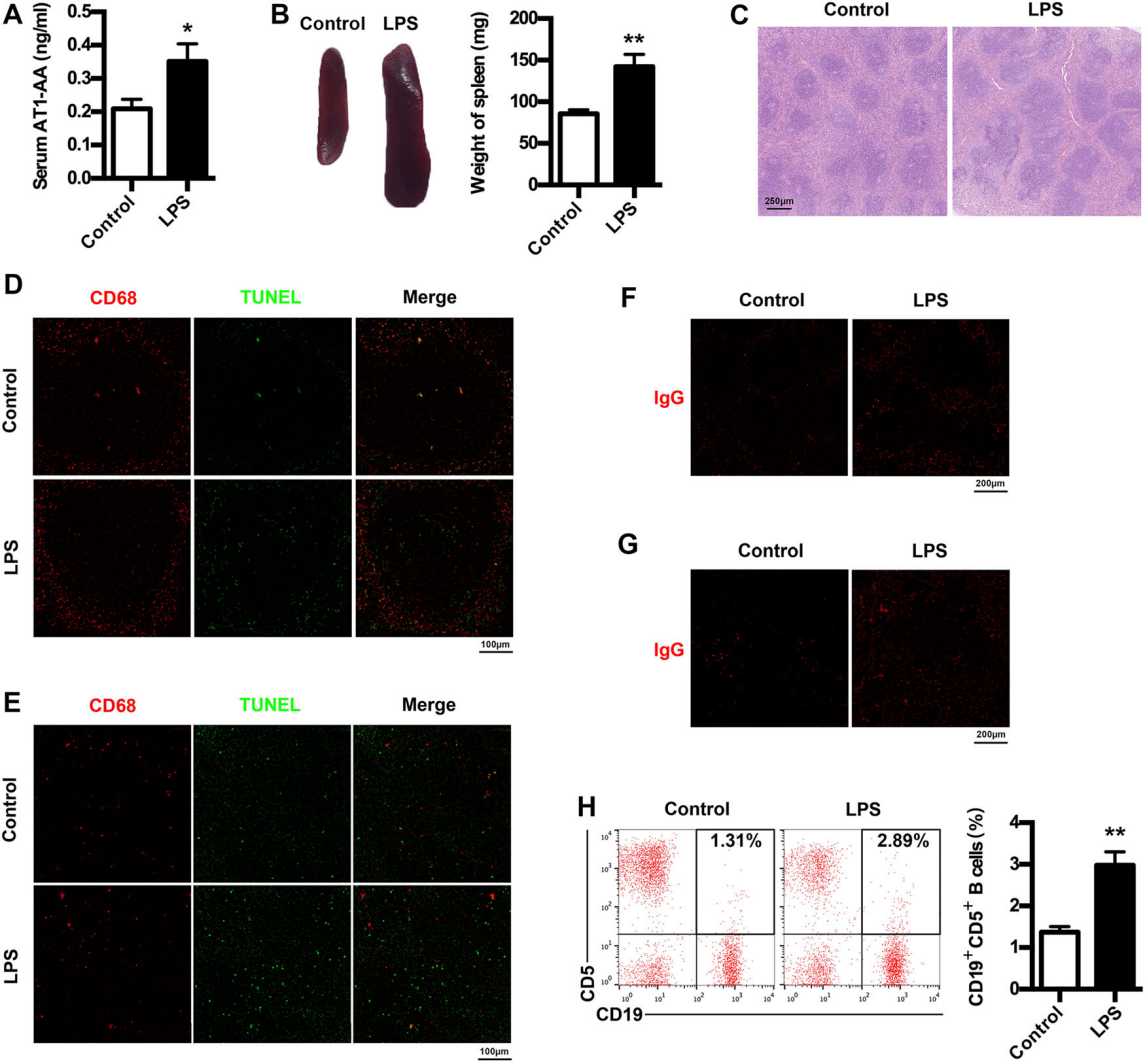
AT1-AA production in PE mice model. **a** Comparison of serum AT1-AA levels between control and PE mice. **b** Comparison of spleen size between control and PE mice. **c** Comparison of spleen pathological features between control and PE mice. Representative H&E staining images of spleen are showed. The bar is 250 μm. **d**, **e** IHC analysis of spleen and lymph node. Sections of spleen (**d**) and lymph node (**e**) were prepared from control and PE mice, and stained with antibody against CD68 (red) and TUNEL (green); staining profiles were merged in the third column. Bar = 100 μm. **f**, **g** IHC analysis of spleen and lymph node. Sections of spleen (**f**) and lymph node (**g**) were prepared from control and PE mice, and stained with antibody against IgG (red). Bar = 200 μm. **h** Comparison of peripheral AT1-AA-producing CD19^+^CD5^+^ B cells between control and PE mice. CD19^+^CD5^+^ B cells in peripheral blood of mice were stained with APC-conjugated Rat Anti-Mouse CD19 and PE-conjugated Rat Anti-Mouse CD5, and subjected to flow cytometry analysis. Results are expressed as means ± SEM (*n* = 7 mice in each group). *p < 0.05 and **p < 0.01 versus control group, two-tailed Student’s *t* test.

To examine AT1-AA production in PE mice, we determined AT1-AA expressions as well as spleen changes, cell apoptosis in spleen and lymph node, IgG production and CD19^+^CD5^+^ B cells percentage. AT1-AA production was markedly elevated in PE mice (Fig. 3a). LPS-induced PE mice developed splenomegaly (Fig. 3b). H&E analysis of spleen indicated that white pulps were greatly enlarged in PE mice (Fig. 3c). LPS treatment resulted in a marked increase in TUNEL-stained dead cells in germinal center of spleen and lymph node (Fig. 3d, e), and apoptotic cells were located outside macrophages expressing CD68 in PE mice. Moreover, increased IgG were found in both spleen and lymph node of PE mice (Fig. 3f, g). Furthermore, peripheral CD19^+^CD5^+^ cells were markedly elevated in PE mice (Fig. 3h). These results suggest the enhancement of AT1-AA production in PE mouse model.

### Caspase-1 deficiency inhibits trophoblast pyroptosis and AT1R antigen exposure in PE mice and trophoblast model

**Fig. 4:**
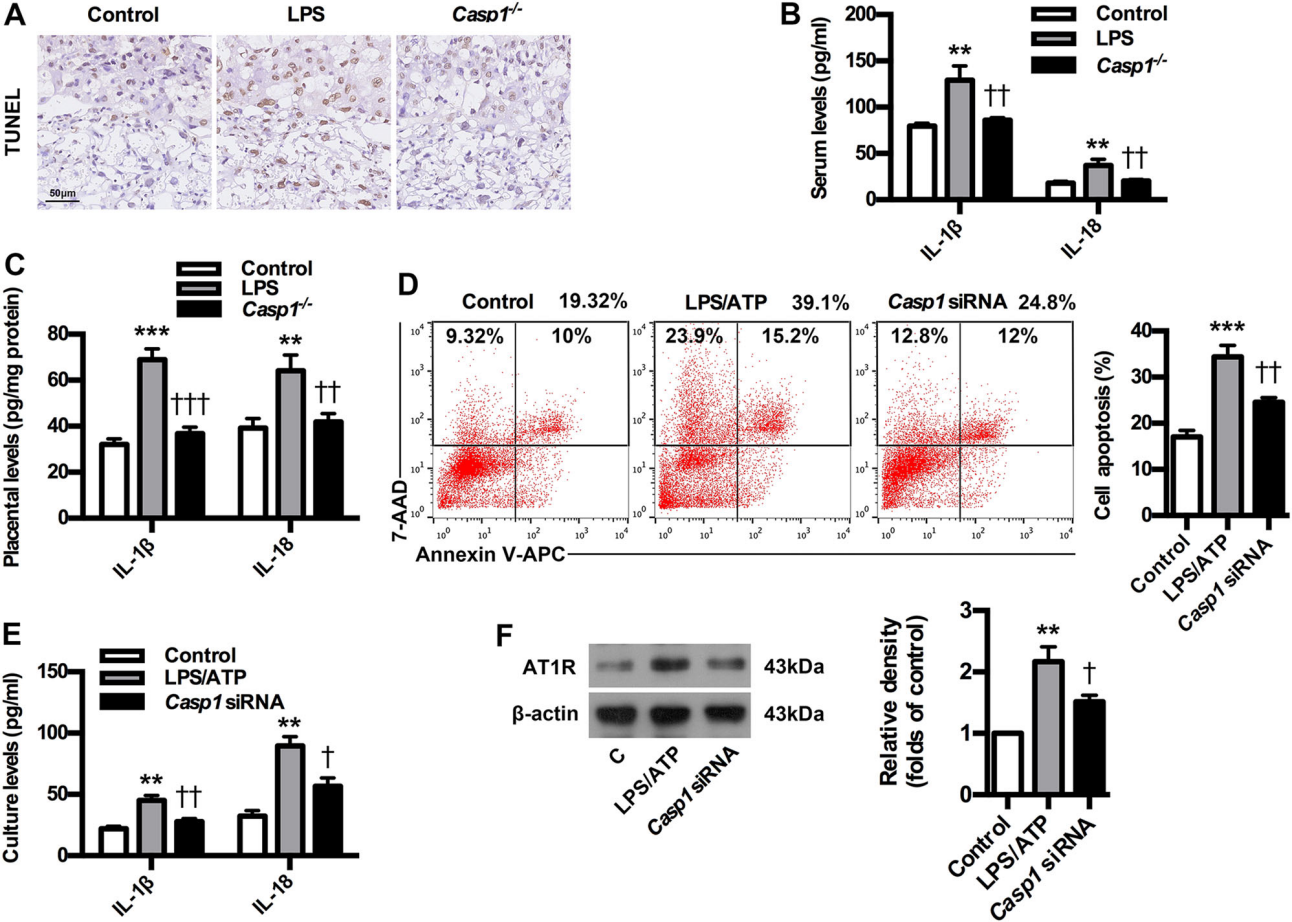
Caspase-1 deficiency inhibits trophoblast pyroptosis and AT1R antigen exposure in PE mice and trophoblast model. **a** Effect of caspase-1 knockout on trophoblast death in PE mice. TUNEL analysis in placenta was shown. Bar = 50 μm. Effect of caspase-1 knockout on IL-1β and IL-18 levels in serum (**b**) and placenta (**c**) of PE mice. IL-1β and IL-18 were detected by ELISA. Results are expressed as means ± SEM (*n* = 7 mice in each group). **P < 0.01 and ***P < 0.001 versus control group, ^††^*P* < 0.01 and ^†††^*P* < 0.001 versus LPS group, one-way ANOVA with S-N-K posttest. **d** Effect of caspase-1 knockdown on trophoblast apoptosis. Cell apoptosis was detected by using flow cytometry. **e** Effect of caspase-1 knockdown on trophoblast IL-1β and IL-18 levels. IL-1β and IL-18 were detected by ELISA. **f** Effect of caspase-1 knockdown on trophoblast AT1R exposure. AT1R was detected by WB. Results are expressed as means ± SEM from three independent experiments. **P < 0.01 and ***P < 0.001 versus control group, ^†^*P* < 0.05 and ^††^*P* < 0.01 versus LPS group, one-way ANOVA with S-N-K posttest.

To confirm the role of caspase-1 in AT1-AA production, a caspase-1 knockout mouse model in C57BL/6 background was constructed. Caspase-1 knockout significantly ameliorated PE-related symptoms of experimental mice (Supplementary Fig. 2a–e). Meanwhile, the absence of caspase-1 resulted in the inhibition of p-P38 and NF-κB expressions in placenta of PE mice, and abrogated the upregulation of sFlt-1, sENG and ET-1 in both serum and placenta of PE mice (Supplementary Fig. 2f–h). These results suggest that the absence of caspase-1 could improve PE-related symptoms in PE mice. Next, we determined whether caspase-1 deficiency could inhibit trophoblast pyroptosis and AT1R exposure by employing PE mice and trophoblast model. As shown in Fig. 4a, caspase-1 knockout inhibited trophoblast death of experimental PE mice. Furthermore, caspase-1 knockout could decrease the expressions of IL-1β and IL-18 in both serum and placenta of PE mice (Fig. 4b, c). Interestingly, the proportions of late apoptotic/necrotic trophoblast cells were downregulated after *Casp1* siRNA treatment compared to LPS/ATP group (Fig. 4d). Moreover, *Casp1* siRNA could suppress the expressions of IL-1β and IL-18 in trophoblast cell culture (Fig. 4e). Importantly, the expressions of AT1-AA specific autoantigen AT1R were inhibited by *Casp1* siRNA (Fig. 4f). These results suggest that caspase-1 deficiency could inhibit trophoblast pyroptosis and AT1R exposure in PE mice and trophoblast model.

### The lack of caspase-1 suppresses AT1-AA production

**Fig. 5:**
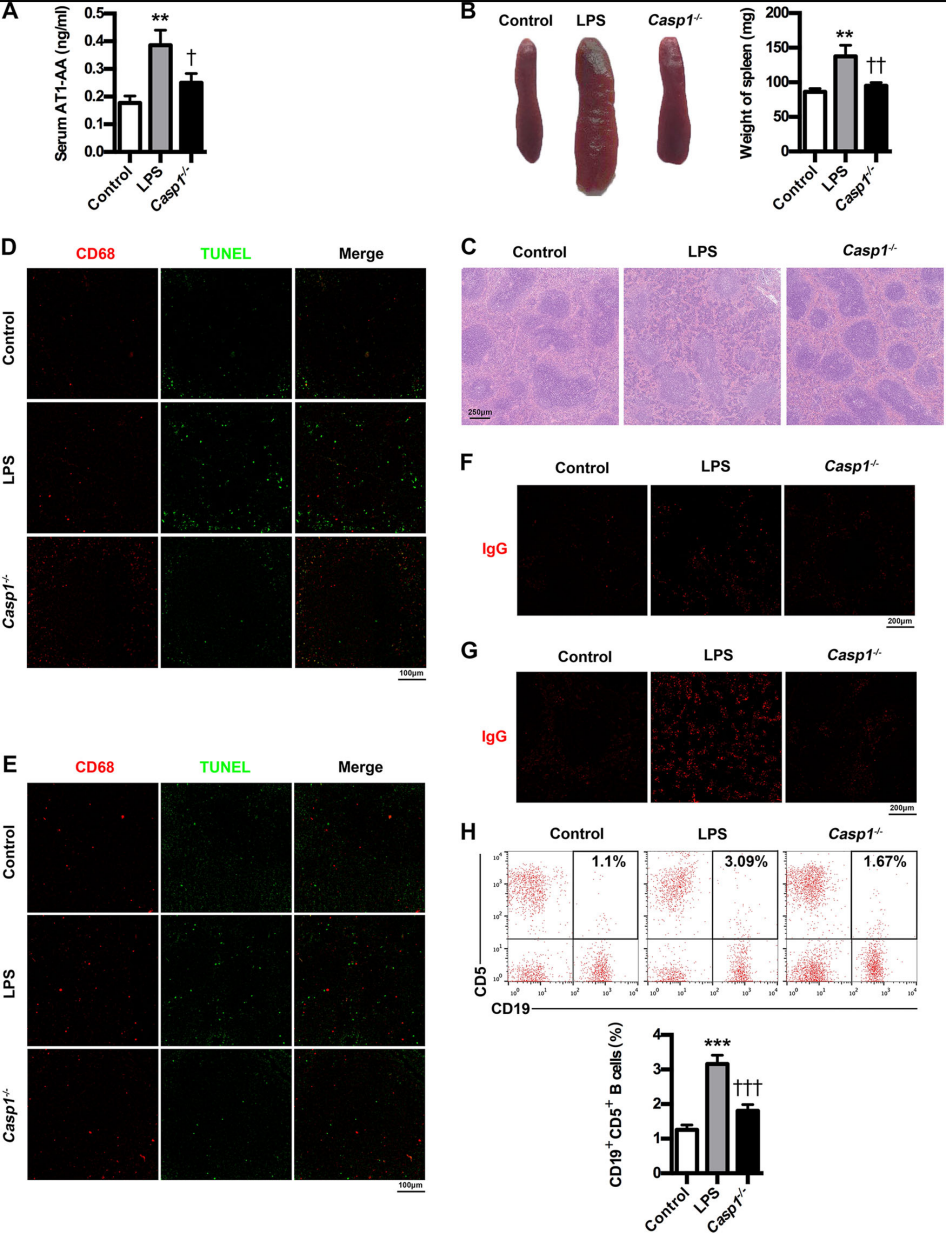
Caspase-1 knockout suppresses AT1-AA production in PE mice. **a** Effect of caspase-1 knockout on AT1-AA expressions in PE mice. **b** Effect of caspase-1 knockout on spleen size in PE mice. **c** Effect of caspase-1 knockout on spleen pathological features in PE mice. Representative H&E staining images of spleen are showed. The bar is 250 μm. **d**, **e** IHC analysis of spleen and lymph node. Sections of spleen (**d**) and lymph node (**e**) were prepared from control, PE and *Casp1*^−/−^ mice, and stained with antibody against CD68 (red) and TUNEL (green); staining profiles were merged in the third column. Bar = 100 μm. **f**, **g** IHC analysis of spleen and lymph node. Sections of spleen (**f**) and lymph node (**g**) were prepared from control, PE and *Casp1*^−/−^ mice, and stained with antibody against IgG (red). Bar = 200 μm. **h** Effect of caspase-1 knockout on peripheral AT1-AA-producing CD19^+^CD5^+^ B cells in PE mice. CD19^+^CD5^+^ B cells in peripheral blood of mice were stained with APC-conjugated Rat Anti-Mouse CD19 and PE-conjugated Rat Anti-Mouse CD5, and subjected to flow cytometry analysis. Results are expressed as means ± SEM (*n* = 7 mice in each group). **P < 0.01 and ***P < 0.001 versus control group, ^†^*P* < 0.05, ^††^*P* < 0.01 and ^†††^*P* < 0.001 versus LPS group, one-way ANOVA with S-N-K posttest.

To further confirm the essential role of caspase-1 in mediating AT1-AA production, we studied the effects of caspase-1 deficiency on AT1-AA expressions as well as spleen changes, cells apoptosis in spleen and lymph node, IgG production and CD19^+^CD5^+^ B cells percentage. As expected, caspase-1 knockout could markedly inhibit AT1-AA production in PE mice (Fig. 5a). Meanwhile, caspase-1 knockout ameliorated spleen size and pathology (Fig. 5b, c). Caspase-1 absence resulted in a marked decrease in TUNEL-stained dead cells in germinal center of spleen and lymph node of PE mice (Fig. 5d, e). Moreover, caspase-1 knockout downregulated IgG in both spleen and lymph node (Fig. 5f, g). Also, caspase-1 knockout could decrease peripheral CD19^+^CD5^+^ cells (Fig. 5h). These results indicate the essential role of caspase-1 in mediating AT1-AA production.

### Caspase-1 activation and AT1-AA expression is associated with LXA_4_ deficiency in human PE

**Fig. 6:**
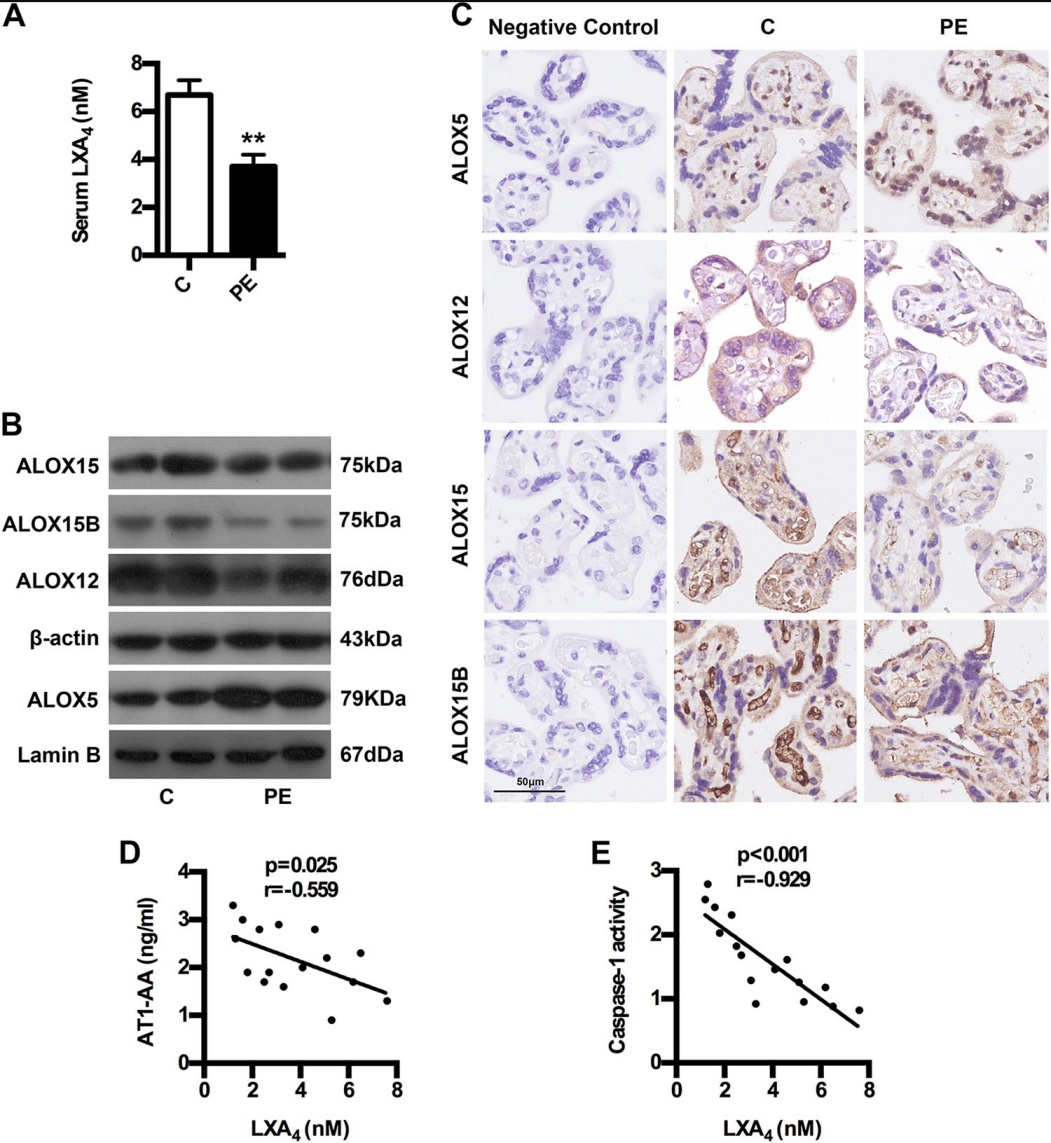
The relationship between LXA_4_ and AT1-AA and caspase-1 in PE patients. **a** Comparison of serum LXA_4_ levels between PE patients and healthy controls. **b**, **c** Comparison of LXA_4_-synthesizing enzymes expressions between PE patients and healthy controls. **b** WB analysis of ALOX5, ALOX12, ALOX15 and ALOX15B in placenta. β-actin from Fig. 1c was reused in Fig. 6b for ease of reference. In Fig. 1c and Fig. 6b, three gels were run, and six blots were performed for detecting ALOX12, ALOX15, ALOX15B and caspase-1. In detail, ALOX12 (76 kDa) and β-actin (43 kDa) proteins were cut from the first gel, electroblotted onto PVDF membrane, and then probed with ALOX12 and β-actin antibodies, respectively. ALOX15 (75 kDa), caspase-1 (45 kDa) and caspase-1 (20 kDa) proteins were cut from the second gel, electroblotted onto PVDF membrane, and then probed with ALOX15 and caspase-1 antibodies, respectively. ALOX15B (75 kDa) protein was cut from the third gel, electroblotted onto PVDF membrane, and then probed with ALOX15B antibody. **c** IHC analysis of ALOX5, ALOX12, ALOX15 and ALOX15B in placenta. Bar = 50 μm. **d**, **e** The correlation between LXA_4_ and AT1-AA and caspase-1 in PE patients. Results are expressed as means ± SEM (*n* = 16 in each group). **p < 0.01 versus control group, two-tailed Student’s *t* test.

The levels of LXA_4_ were firstly measured to confirm its change in human PE. As shown in Fig. 6a, LXA_4_ levels in PE were strikingly downregulated compared to controls. To confirm the abnormality of LXA_4_ biosynthesis, we detected the expressions of ALOX5, ALOX12, ALOX15 and ALOX15B. As shown in Fig. 6b, c, the expressions of ALOX12, ALOX15 and ALOX15B were downregulated in PE placenta compared with controls, while placental ALOX5 was abnormally activated, which was responsible for LXA_4_ deficiency in PE. But were caspase-1 activation and AT1-AA expression really associated with LXA_4_ deficiency in PE? Correlation analysis was employed to further confirm the role of LXA_4_ in caspase-1 activation and AT1-AA expression. It was found that caspase-1 activities and AT1-AA expressions were negatively correlated with LXA_4_ levels (Fig. 6d, e). These clinical data suggest that caspase-1 activation and AT1-AA expression is associated with LXA_4_ deficiency in PE.

### LXA_4_ suppresses caspase-1 activation, trophoblast pyroptosis and AT1R exposure as well as enhance phagocytosis of apoptotic trophoblasts by macrophages

**Fig. 7:**
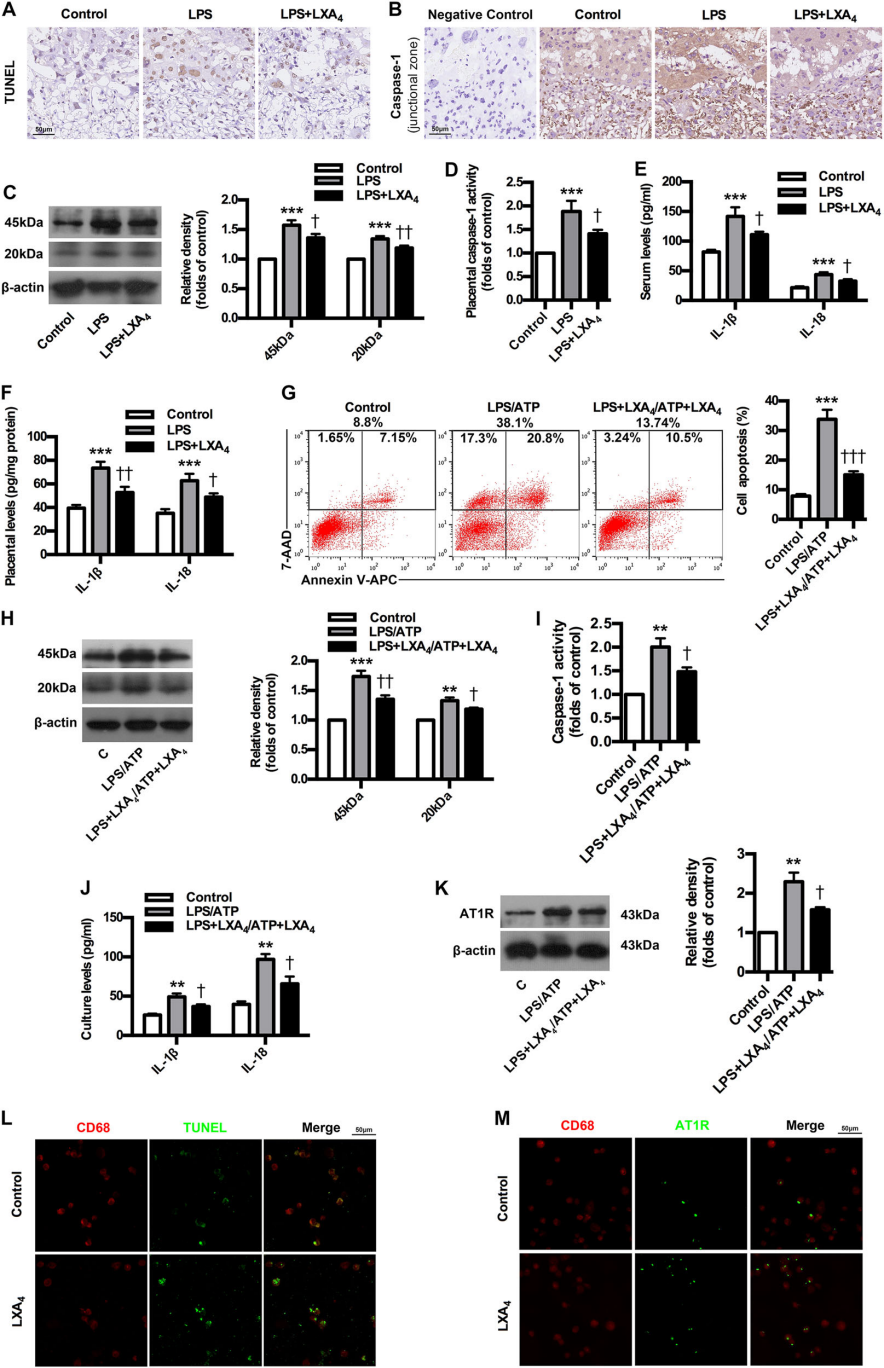
LXA_4_ inhibits caspase-1 activation, trophoblast pyroptosis and AT1R exposure as well as enhance phagocytosis of apoptotic trophoblasts by macrophages. **a** Effect of LXA_4_ on trophoblast death in PE mice. TUNEL analysis in placenta was shown. Bar = 50 μm. **b**, **c** Effect of LXA_4_ on placental caspase-1 expression in PE mice. IHC (**b**) and WB (**c**) analysis of caspase-1 in placenta were shown. The histogram represents means ± SEM of the densitometric scans for protein bands (*n* = 7 mice in each group), normalized by comparison with β-actin and expressed as a percentage of Control. Bar = 50 μm. **d** Effect of LXA_4_ on placental caspase-1 activity in PE mice. Effect of LXA_4_ on IL-1β and IL-18 levels in serum (**e**) and placenta (**f**) of PE mice. IL-1β and IL-18 were detected by ELISA. Results are expressed as means ± SEM (*n* = 7 mice in each group). ***P < 0.001 versus control group, ^†^*P* < 0.05 and ^††^*P* < 0.01 versus LPS group, one-way ANOVA with S-N-K posttest. **g** Effect of LXA_4_ on trophoblast apoptosis. Cell apoptosis was detected by using flow cytometry. **h** Effect of LXA_4_ on trophoblast caspase-1 expression. Caspase-1 expression was detected by WB. **i** Effect of LXA_4_ on trophoblast caspase-1 activity_._ **j** Effect of LXA_4_ on trophoblast IL-1β and IL-18 levels. IL-1β and IL-18 were detected by ELISA. **k** Effect of LXA_4_ on trophoblast AT1R exposure. AT1R was detected by WB. Results are expressed as means ± SEM from three independent experiments. **P < 0.01 and ***P < 0.001 versus control group, ^†^*P* < 0.05, ^††^*P* < 0.01 and ^†††^*P* < 0.001 versus LPS group, one^-^way ANOVA with S-N-K posttest. **l**, **m** Effect of LXA_4_ on phagocytosis of apoptotic trophoblasts by macrophages. IF analysis of phagocytosis of apoptotic trophoblasts (**l**) and AT1R (**m**) by macrophages were shown. Slides of macrophages phagocytosis of apoptotic trophoblasts were prepared from control and LXA_4_ group, and stained with antibody against CD68 (red) and TUNEL (green), or antibody against CD68 (red) and AT1R (green); staining profiles were merged in the third column. Bar = 50 μm.

To confirm the regulatory roles of LXA_4_ in the process of AT1-AA production, LPS-induced PE mice were treated with LXA_4_. As expected, LXA_4_ administration effectively alleviated PE-related symptoms of experimental PE mice (Supplementary Fig. 3a–e). Meanwhile, LXA_4_ inhibited the expressions of p-P38 and NF-κB in placenta of PE mice, and abrogated the upregulation of sFlt-1, sENG and ET-1 in both serum and placenta of PEE mice (Supplementary Fig. 3f–h). After demonstrating the protective roles of LXA_4_ on PE, we next determined whether LXA_4_ could inhibit caspase-1 activation, trophoblast pyroptosis and AT1R exposure. As shown in Fig. 7a, LXA_4_ treatment inhibited trophoblast death. LXA_4_ downregulated caspase-1 expressions in placenta of PE mice (Fig. 7b, c). In line with this, LXA_4_ suppressed placental caspase-1 activity in PE mice (Fig. 7d). Furthermore, LXA_4_ could decrease the levels of IL-1β and IL-18 in both serum and placenta of PE mice (Fig. 7e, f). Interestingly, the proportions of late apoptotic/necrotic trophoblast cells were significantly downregulated by LXA_4_ compared to LPS/ATP group (Fig. 7g). LXA_4_ could also inhibit trophoblast caspase-1 expressions stimulated by LPS/ATP (Fig. 7h). In line with this, LXA_4_ could suppress trophoblast caspase-1 activity (Fig. 7i). Moreover, LXA_4_ could decrease the levels of IL-1β and IL-18 in trophoblast cell cultures (Fig. 7j). Importantly, the expressions of AT1-AA specific autoantigen AT1R were inhibited by LXA_4_ (Fig. 7k). These results suggest that LXA_4_ could inhibit caspase-1 activation, trophoblast pyroptosis and AT1R exposure during AT1-AA production. To observe the effects of LXA_4_ on macrophages in phagocytosing dead trophoblasts and AT1R, IF analysis was employed. Resultedly, LXA_4_ greatly improved the efficiency of macrophages in phagocytosing dead trophoblasts and AT1R (Fig. 7l, m).

### LXA_4_ inhibits AT1-AA production in PE mice

**Fig. 8:**
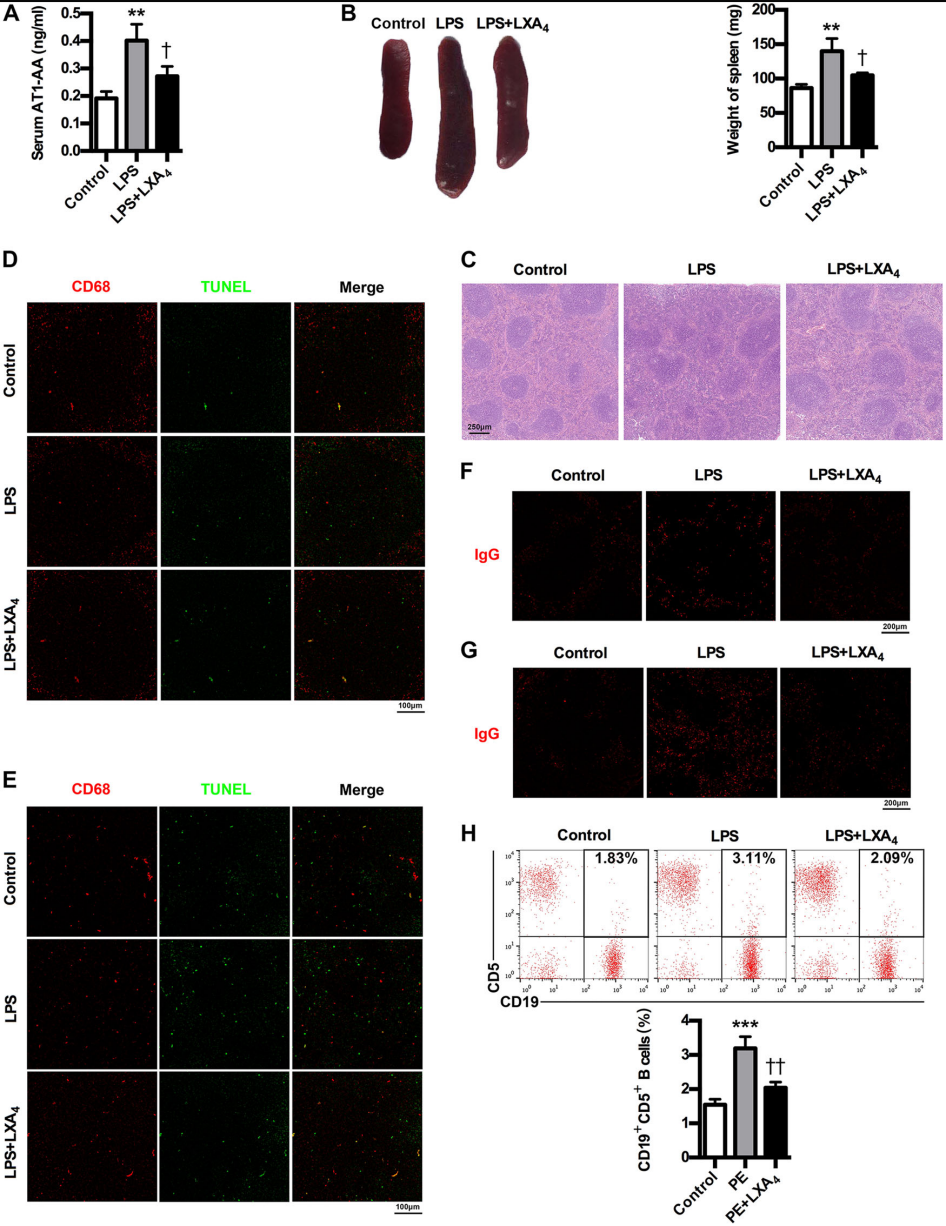
LXA_4_ suppresses AT1-AA production in PE mice. **a** Effect of LXA_4_ on AT1-AA expressions in PE mice. **b** Effect of LXA_4_ on spleen size in PE mice. **c** Effect of LXA_4_ on spleen pathological features in PE mice. Representative H&E staining images of spleen are showed. The bar is 250 μm. **d**, **e** IHC analysis of spleen and lymph node. Sections of spleen (**d**) and lymph node (**e**) were prepared from control, PE and LXA_4_ mice, and stained with antibody against CD68 (red) and TUNEL (green); staining profiles were merged in the third column. Bar = 100 μm. **f, g** IHC analysis of spleen and lymph node. Sections of spleen (**f**) and lymph node (**g**) were prepared from control, PE and LXA_4_ mice, and stained with antibody against IgG (red). Bar = 200 μm. **h** Effect of LXA_4_ on peripheral CD19^+^CD5^+^ B cells in PE mice. CD19^+^CD5^+^ B cells in peripheral blood of mice were stained with APC-conjugated Rat Anti-Mouse CD19 and PE-conjugated Rat Anti-Mouse CD5, and subjected to flow cytometry analysis. Results are expressed as means ± SEM (*n* = 7 mice in each group). **P < 0.01 and ***P < 0.001 versus control group, ^†^*P* < 0.05 and ^††^*P* < 0.01 versus LPS group, one-way ANOVA with S-N-K posttest.

To further confirm the important roles of LXA_4_ in inhibiting AT1-AA production, we investigated the effects of LXA_4_ on AT1-AA expressions as well as spleen changes, cells apoptosis and phagocytic activity of macrophages in spleen and lymph node, IgG production and CD19^+^CD5^+^ B cells percentage in PE mice. It has showed that LXA_4_ could markedly inhibit AT1-AA production in PE mice (Fig. 8a). Meanwhile, LXA_4_ ameliorated spleen size and pathology (Fig. 8b, c). Moreover, LXA_4_ treatment resulted in a marked decrease in TUNEL-stained dead cells and a marked increase in the phagocytosis of dead cells by macrophages in germinal center of spleen and lymph node of PE mice (Fig. 8d, e). Furthermore, LXA_4_ could downregulate IgG in both spleen and lymph node (Fig. 8f, g). Importantly, LXA_4_ could decrease peripheral CD19^+^CD5^+^ B cells (Fig. 8h). These results confirm the important roles of LXA_4_ in inhibiting AT1-AA production.

## Discussion

We demonstrate for the first time that caspase-1 activation is involved in AT1-AA production in PE, and LXA_4_ could suppress AT1-AA production via regulating caspase-1 as well as enhancing phagocytosis of apoptotic trophoblast cells by macrophages. Our data indicate that caspase-1 may serve as an emerging key therapeutic target for attenuating AT1-AA production in PE and LXA_4_ could protect PE patients from AT1-AA via modulating caspase-1.

This study revealed several novel findings regarding the relationship between caspase-1, AT1-AA and LXA_4_. Foremost, PE patients and mice model developed AT1-AA associated with caspase-1 activation. Furthermore, caspase-1 activation could promote AT1-AA production via inducing trophoblast pyroptosis and AT1R exposure. Lastly, LXA_4_ could inhibit AT1-AA production via regulating caspase-1. Hence, this study is the first to report caspase-1 as a key target in mediating AT1-AA production and LXA_4_ as a protective regulator in inhibiting AT1-AA via caspase-1.

Although studies of inflammasome pathways in PE are limited, it has been showed that placental inflammation in PE could be activated by NLRP3 inflammasome, suggesting contribution of NLRP3 to the harmful placental inflammation and PE^31^. Moreover, Weel et al. showed increased expressions of NLRP3 and caspase-1 in PE placentas^32^. The present study aimed to investigate whether AT1-AA production is associated with caspase-1 activation and trophoblast pyroptosis. Resultedly, the expressions and activity of caspase-1 in PE placentas were significantly higher than those of normal pregnancies. Furthermore, placental levels of IL-1β and IL-18 in PE increased significantly compared to controls. Meanwhile, trophoblast death was exacerbated in PE. As expected, we showed higher expressions of AT1-AA in PE serum compared to normal pregnancies, which is in line with previous studies^6,10,33^. Of importance, our data demonstrated significant correlations between AT1-AA and caspase-1, IL-1β, IL-18 and LDH in PE. To summarize, these clinical data strongly support an important role for caspase-1-mediated pyroptosis in AT1-AA production.

To validate the clinical data in animal model, PE model and caspase-1 knockout model were constructed in mice. Here, classic hallmarks of human PE, including hypertension, proteinuria, fetus restriction, placental oxidative damage and renal impairment, appeared in PE mice, while caspase-1 knockout could improve PE-related symptoms. A common pathophysiology of PE is endothelial dysfunction and angiogenic/angiostatic imbalance, involving sFlt-1, sEng and ET-1^2,4,6,7,13^. Accordingly, we showed higher expressions of sFlt-1, sEng and ET-1 in serum and placenta from PE mice, while the absence of caspase-1 abrogated the upregulation of sFlt-1, sENG and ET-1. Activation of p38 and NF-kB signaling pathways plays important roles in mediating the release of inflammatory mediators and antiangiogenic factors (sFlt-1, sEng and ET-1), which stimulate systemic inflammatory reactions and endothelial dysfunction that manifests PE symptoms^27,34–36^. We demonstrated in the present study enhanced expressions of p-P38 and NF-κB in placenta of PE mice, while p-P38 and NF-κB were inhibited by caspase-1 knockout. Together, these results support the essential role of caspase-1 in determining PE pathogenesis. By employing the PE model, we confirmed that trophoblast death was enhanced, and placental caspase-1 was activated in PE mice. Interestingly, caspase-1 knockout inhibited trophoblast death, suggesting the involvement of caspase-1 in trophoblast pyroptosis. Pyroptosis is induced by two distinct stimuli, exogenous PAMPs and endogenous DAMPs. Thus, to elucidate the mechanisms determining caspase-1 in AT1-AA production in vitro, we utilized LPS and ATP as PAMP and DAMP, respectively. As expected, the stimulation of trophoblast cells with LPS and ATP could induce pyroptosis features, including trophoblast death, caspase-1 activation and IL-1β and IL-18 increase. Meanwhile, LPS/ATP could stimulate the release of AT1-AA specific autoantigen AT1R. However, *Casp1* siRNA could suppress trophoblast death, IL-1β and IL-18 expressions and AT1R exposure, supporting the involvement of caspase-1 in trophoblast pyroptosis and AT1R exposure. Macrophages engulf apoptotic cells generated in the germinal center^15^, while defective phagocytosis of apoptotic cells may lead to excessive appearance of autoantigens and stimulate autoantibody production. Germinal centers contain macrophages called TBMs, specifically expressing CD68, which are present not only in the spleen but also in the lymph nodes^15^. In the study, a marked increase in the number of TUNEL-stained dead cells appeared in the germinal center of spleen and lymph node of PE mice. The reason for aggravated accumulation of dead cells in spleen and lymph node was partially caused by exacerbated apoptosis and partially caused by inefficient phagocytosis of apoptotic cells by TBMs. B cells might be involved in AT1-AA production in PE, and a specific mature B-cell subtype, CD19^+^CD5^+^ B cells, are demonstrated to be responsible for AT1-AA production^4,37,38^. Moreover, CD19^+^CD5^+^ B cells were confirmed to be enriched in peripheral blood of PE patients^4^. Indeed, in the study, peripheral CD19^+^CD5^+^ cells were markedly elevated in PE mice, and, meanwhile, increased number of IgG were found not only in spleen but also in lymph node of PE mice. Importantly, caspase-1 knockout could markedly inhibit AT1-AA production as well as ameliorate cell death in the germinal center and downregulate peripheral CD19^+^CD5^+^ cells and IgG in spleen and lymph node. Together, relying on the results gathered by using mice model and trophoblast model, the study provides evidence supporting the involvement of caspase-1 activation in trophoblast pyroptosis, AT1R exposure and AT1-AA production in PE. To our knowledge, this is an original discovery suggesting a prominent role for caspase-1 in AT1-AA production and PE pathogenesis.

The efficient nonimmunogenic clearance of ACs is crucial to dispose self-antigens and to maintain self-tolerance. A previous report showed that ALOX15 orchestrates the clearance of ACs and maintains immunologic tolerance^30^. ALOX15 can contribute to the generation of LXA_4_, which, in turn, is identified as important factor initiating the resolution of inflammation and has been implicated in the removal of ACs^30^. Here, we showed LXA_4_ decrease in PE patients. Meanwhile, we identified the expressions of LXA_4_-biosynthesis enzymes ALOX5, ALOX15, ALOX15B and ALOX12 in PE patients, and found the downregulation of ALOX12, ALOX15 and ALOX15B but the activation of ALOX5. ALOX5 is the key enzyme for both leukotrienes (LTs) and lipoxins (LXs) biosynthesis. Upon stimulation, it translocates to the nuclear membrane to form enzyme complex, which converts AA to LTs^39^. ALOX5 activation suggests possible class switching from LXs to LTs production, which has been further confirmed by our previous report that ALOX5 activation contributed to enhanced LTB_4_ while decreased LXA_4_ biosynthesis in rat placenta^27^. Thus, the downregulation of ALOX12, ALOX15 and ALOX15B while the activation of ALOX5 were responsible for LXA_4_ deficiency in PE. Importantly, we demonstrated an association between LXA_4_ decrease and caspase-1 activation and AT1-AA production in human PE. By employing PE mice model, we further confirmed that LXA_4_ could suppress trophoblast death, caspase-1 activation and IL-1β and IL-18 expressions, providing the evidence supporting the regulation of LXA_4_ on caspase-1 activation and trophoblast pyroptosis. In line with this, LXA_4_ exhibits protective effects on the PE animal model. LXA_4_ could also inhibit the expressions of sFlt-1, sENG and ET-1 as well as p-P38 and NF-κB in PE mice. Moreover, LXA_4_ could inhibit trophoblast death, caspase-1 activation, IL-1β and IL-18 expressions and AT1R exposure in vitro, provide further evidence supporting the regulatory roles of LXA_4_ on caspase-1 activation, trophoblast pyroptosis and AT1R exposure. We next elucidated the effects of LXA_4_ on macrophages efficiency in engulfing dead trophoblast and AT1R autoantigen. The results indicated that LXA_4_ could promote the efficiency of macrophages to engulf dead trophoblast and AT1R. Accordingly, LXA_4_ could indeed inhibit AT1-AA production. Meanwhile, LXA_4_ could ameliorate spleen pathology and macrophages phagocytosis as well as downregulate peripheral CD19^+^CD5^+^ cells and IgG in spleen and lymph node in PE mice. The present study has demonstrated for the first time an additional function of endogenous LXA_4_ to suppress autoantibody AT1-AA by inhibiting caspase-1 activation, trophoblast pyroptosis and AT1R exposure as well as enhancing macrophages phagocytosis of dead trophoblast cells.

In conclusion, the present study demonstrated the relevance of caspase-1 to AT1-AA in PE by using clinical data. Relying on the results gathered by using mice model, knockout model and trophoblast model, the study confirmed that caspase-1 is involved in AT1-AA production. By employing PE animal model and trophoblast model, the study further indicated that LXA_4_ could suppress AT1-AA production via modulating caspase-1. The study proposes a common mechanism underlying AT1-AA production, providing new insights into the understanding of PE. Our data suggest that avoidance of PAMPs and DAMPs exposure and administration of LXA_4_ might be potential strategies to prevent AT1-AA production, and also contribute to develop other potential therapeutic strategies in preventing PE by targeting caspase-1. This finding may provide a new direction for the drug design of proresolving lipid mediator utilizing LXA_4_ as a lead compound with a dual action on pyroptosis and phagocytosis to inhibiting autoantibody production in PE or other autoimmune diseases.

## Materials and methods

### Reagents and antibodies

LPS (Escherichia coli serotype O127:B8) was from Sigma Aldrich (Allentown, PA, USA). ATP disodium salt and phorbol 12-myristate 13-acetate (PMA) were from MedChemExpress (Monmouth Junction, NJ, USA). A stock solution of LXA_4_ (Cayman Chemical, Ann Arbor, MI, USA) was stored at −80 °C until being diluted in sterile 0.9% saline or cell culture medium immediately before use. Anti-NF-κB p65, -ALOX12, -ALOX15, -ALOX15B, -AT1R, -β-actin, and -Lamin B antibodies were from Santa Cruz Biotechnology (Santa Cruz, CA, USA). Anti phospho-P38 and P38 antibodies were obtained from Cell Signaling Technology (Danvers, MA, USA). Anti-Caspase-1 antibody was from Abcam Company (Cambridge, UK). Anti-CD68 antibody was from Proteintech Group, Inc. (Wuhan, China). APC-conjugated Rat Anti-Mouse CD19 and PE-conjugated Rat Anti-Mouse CD5 were from BD Biosciences (San Diego, CA, USA). Mouse ALOX5 monoclonal antibody (BD Biosciences) was used for western blotting (WB), whereas rabbit ALOX5 polyclonal antibody (Cayman Chemical) was used for immunohistochemistry (IHC). RIPA Lysis Buffer and Nuclear and Cytoplasmic Protein Extraction Kit were from Beyotime Institute of Biotechnology (Shanghai, China). BCA protein assay kit was from Pierce (Rockford, IL, USA).

### Patient samples

Blood samples were obtained from 32 women: 16 women with PE defined by hypertension (systolic and diastolic blood pressures higher than 140/90 mmHg) and proteinuria (0.3 g/24 h)^40^ were recruited from the Puai Hospital, Tongji Medical College, Huazhong University of Science and Technology, China; as a comparative group, 16 pregnant women were originally selected with characteristics similar to those presented by the preeclamptic patients, including body mass index, gestational age, eliminated high blood pressure, kidney disease, diabetes, and so on. Blood and placenta samplings were obtained from diagnosed patients after informed consent and approved by the Ethical Committee of the Medical Faculty of Tongji Medical College, Huazhong University of Science and Technology in accordance with the Declaration of Helsinki.

The patients had not taken any medications before specimen collection. Blood extracted from the pregnant women stood for 20 min at room temperature. After centrifugation, serum was collected and stored at −80 °C until use. The serums were used to detect the levels of AT1-AA and LXA_4_.

### Animals and experimental protocol

C57BL/6N mice (8–10-week old, weighing 18–22 g) were purchased from the Beijing Vital River Laboratory Animal Technology Co., Ltd Caspase-1 knockout mice (*Casp1*^*−/−*^*)* B6N.129S2-*Casp1*^*tm1Flv*^/J (No.016621) were purchased from The Jackson Laboratory. Animals were housed individually in plastic cages with wood chips as bedding under pathogen-free conditions, in a controlled environment of temperature at 20–25 °C and 12 h cycles of light and dark. Mice were fed a standard laboratory diet and water *ad libitum*. Pregnancy was obtained by mating female mice with fertile male mice at a ratio of 2:1 overnight. Daily vaginal smears were observed, and appearance of spermatozoa in vaginal smear was defined as gestational day (GD) 1. All animal work was conducted according to the recommendations in the Guide for the Care and Use of Laboratory Animals of the National Institutes of Health. All studies involving mice were approved by Animal Care and Use Committee of Huazhong University of Science and Technology.

#### Experimental protocol 1

Pregnant mice were randomly divided into control group (*n* = 7) and LPS group (*n* = 7). Experimental PE model was induced by infusion of LPS (1 μg/kg body weight) in 200 μl of sterile saline through an infusion pump into the tail vein (infusion rate, 200 μl/h) on GD 13 according to our initial experiments and previous reports^28,41^. Normal pregnant control mice were infused with equal saline.

#### Experimental protocol 2

Pregnant mice were randomly divided into control group (*n* = 7), LPS group (*n* = 7) and *Casp1*^*−/−*^ group (*n* = 7). Experimental PE model was induced according to above *Experimental protocol 1* in LPS and *Casp1*^*−/−*^ groups, while controls were infused with equal saline.

#### Experimental protocol 3

Pregnant mice were randomly divided into control group (*n* = 7), LPS group (*n* = 7) and LPS + LXA_4_ group (*n* = 7). Experimental PE model was induced according to above *Experimental protocol 1* in LPS and LPS + LXA_4_ groups, while controls were infused with equal saline. After 30 min, mice in LPS + LXA_4_ group were injected i.p. with LXA_4_ (10 μg/kg), whereas mice in control and LPS groups were injected with equal amounts of vehicle.

### Cell culture and treatment

Human first-trimester trophoblast cell line HTR-8/SVneo and human monocytic cell line THP-1 were obtained from American Type Culture Collection (USA). Cells were cultured at 37 °C in a humidified atmosphere with 5% CO_2_ in 1640 medium supplemented with 10% heat-inactivated fetal bovine serum, 25 mM HEPES, 100 U/mL penicillin G and 100 U/mL streptomycin. Then, HTR-8/SVneo cells were primed with 1 μg/ml LPS for 24 h in the absence or presence of LXA_4_ (100 nM), and treated with 5 mM ATP for 24 h in the absence or presence of LXA_4_ (100 nM) for different assays.

### Caspase-1 shRNA transfection experiments

For knockdown studies of trophoblast caspase-1 RNA, HTR-8/SVneo cells were cultured in 6- or 12-well plates until 60% confluent. shRNA vector (GV102) of *Casp1* sequence (*Casp1*-RNAi, 5′-CACGTCTTGCTCTCATTAT-3′) was designed, synthesized, and labeled with GFP (GeneChem, Inc., Shanghai). shRNA vector (GV102) of scrambled sequence (5′-TTCTCCGAACGTGTCACGT-3′) was used as negative control and labeled with GFP (GeneChem, Inc., Shanghai). For each of the shRNA constructs, the HTR-8/SVneo cells were transfected using Lipofectamine-2000 reagent according to the routine methods established in our lab, after which these cells were utilized for different assay 24 h later.

### Affinity purification and determination of AT1-AA

We isolated the IgG fraction from human serum and performed affinity purification of AT1-AA as previously described^9^. Then, we evaluated the concentrations of AT1-AA in serum of pregnant women or mice by employing an ELISA method.

### Measurement of systolic blood pressure (SBP)

At the indicated time (initial nonpregnant status, GD11-12 and GD19), the SBP was determined in conscious, restrained pregnant mice. An automated system with a photoelectric sensor linked to a dual-channel recorder (BP-98A, Softron, Japan), tail cuff and sphygmomanometer was used to obtain indirect blood pressure measurements, which have been previously demonstrated to be closely correlated with direct arterial measurements^42^. The measurements were repeated three times for each mouse, with the mean value recorded.

### Determination of urinary albumin excretion

For 24-h urine collection, on GD11-12 and GD18-19, the pregnant mice were placed in metabolic cages. To avoid contaminating the collected urine, rats were restricted from food; however, they were allowed free access to water. To avoid the adverse effects of fasting, rats were fed in other cages for 30 min every 6 h. Urine samples were centrifuged at 3000 rpm for 20 min at room temperature, and the supernatant was collected for urinary albumin analysis. Urine protein concentrations were determined by using pyrogallol red in an automatic biochemical analyzer (ADVIA 2400 Chemistry System, Siemens Medical Solutions Diagnostics, NY, USA).

### Measurement of thiobarbituric acid reactive substances (TBARS)

Placental TBARS was measured by using a commercially available kit (QuantiChrom^TM^ TBARS Assay Kit, DTBA-100) according to manufacturer’s instruction (BioAssay Systems, USA). Briefly, placentas (~20 mg) were placed into 200 μL ice-cold phosphate-buffered saline with protease inhibitors. The tissues were first homogenized thoroughly and then sonicated for 20 s on ice. Samples were then centrifuged at 3000 rpm for 10 min at 4 °C. Twenty microliters aliquot was removed for protein analysis. The resultant absorbances were read at 535 nm. TBARS levels were expressed as nmol/mg protein.

### TUNEL assay

For the TUNEL assay, cell death was determined using a TUNEL staining kit (Roche Diagnostics, Indianapolis, IN, USA). Briefly, sections of paraffin-embedded placental tissues were deparaffinized and subjected to antigen retrieval. Then, the sections were incubated with a mixture of TdT and fluorescence-labeled nucleotides. The prepared sections were examined with NanoZoomer S360 by NZAcquire (HAMAMATSU, Japan). The data were analyzed by NanoZoomer Digital Pathology (NDP.view 2).

### H&E, IHC and Immunofluorescence (IF) of tissue

For histological evaluation, placenta and kidney were fixed in 4% neutral-buffered polyformaldehyde overnight at room temperature. Tissues were infiltrated and embedded in paraffin. H&E staining was performed by standard techniques on 4-μm paraffin sections of placenta and kidney specimens for conventional morphological evaluation with NanoZoomer S360 by NZAcquire (HAMAMATSU, Japan). The data were analyzed by NanoZoomer Digital Pathology (NDP.view 2).

For assessment of ALOXs expression in human placenta and caspase-1 expression in mice placenta, regular IHC assay was performed. Briefly, 4 μm paraffin sections of specimens were cut, and after deparafinization, antigen retrieval was performed in sodium citrate solution (pH 6.0). Sections were incubated with different primary antibody at 4 °C overnight. Sections were then washed and incubated with secondary antibody for 45 min at room temperature. The sections were subsequently incubated with 3,3’-diaminobenzidine tetrahydrochloride (DAB) substrate, lightly counterstained with hematoxylin, dehydrated, and mounted. The prepared sections of IHC staining were examined with NanoZoomer S360 by NZAcquire (HAMAMATSU, Japan). The data were analyzed by NanoZoomer Digital Pathology (NDP.view 2).

For assessment of cell death accumulation and IgG production in spleen and lymph node, regular IF assay was performed. Briefly, 4 μm paraffin sections of specimens were cut, and after deparafinization, antigen retrieval was performed. Sections were then stained with IgG antibody, or subjected to CD68 staining and TUNEL assay. Fluorescence images were processed using Pannoramic MIDI by pannoramic scanner (3D HISTECH, Hungary). The data were analyzed by Caseviewer software.

### WB

Total and nuclear proteins were extracted using RIPA Lysis Buffer and Nuclear and Cytoplasmic Protein Extraction Kit, respectively. Protein concentrations were determined using a BCA protein assay kit. Equal amounts of protein (40 μg) were then subjected to 12% SDS- PAGE and transferred to PVDF membranes (Millipore). Different primary antibodies were incubated with the membranes overnight at 4 °C. β-actin and Lamin B served as internal control of total and nuclear proteins, respectively. The bound antibody was detected by an enhanced chemiluminescence kit (Thermo Scientific Pierce) on X-ray film.

### Determination of caspase-1 and LDH activity

The caspase-1 activation was assayed by using a commercially available kit (Caspase 1 Activity Assay Kit, C1102) according to manufacturer’s instruction (Beyotime Institute of Biotechnology, Shanghai, China). LDH activities in human placenta were determined for the evaluation of trophoblast death. Briefly, placentas were placed into ice-cold phosphate-buffered saline with protease inhibitors. The tissues were first homogenized thoroughly by electric homogenizer on ice. Samples were then centrifuged at 3000 rpm for 10 min at 4 °C. LDH activities in placental extracts were detected in an automatic biochemical analyzer (ADVIA 2400 Chemistry System, Siemens Medical Solutions Diagnostics, NY, USA).

### Enzyme-linked immunosorbent assay (ELISA)

We determined the concentrations of sFlt-1, sEng and ET-1 in mouse serum and placental tissue and IL-1β and IL-18 in serum, placenta and cell culture with commercial ELISA kits (R&D Systems, Minneapolis, MN, USA) according to the manufacturer’s instructions.

### Determination of LXA_4_

Levels of LXA_4_ in the human serum were assessed by assay kit according to manufacturers’ protocols (Neogen Corporation, Lansing, MI, USA).

### Flow cytometry analysis

CD19^+^CD5^+^ B cells in the peripheral blood of mice were stained with APC-conjugated Rat Anti-Mouse CD19 and PE-conjugated Rat Anti-Mouse CD5, and subjected to flow cytometry analysis (BD FACS Calibur) to examine the percentage of AT1-AA-producing CD19^+^CD5^+^ B cells. Cultured trophoblast cells were stained with 7-AAD and APC-labeled Annexin V (BD Pharmingen) according to the manufacturer’s instructions, and subjected to flow cytometry analysis (BD FACS Calibur) to examine trophoblast cell apoptosis. The data were analyzed by FlowJo software.

### Phagocytosis assay of dead trophoblasts and AT1R antigens

For the preparation of dead trophoblast cells, HTR-8/SVneo cells were primed with 1 μg/ml LPS for 24 h, and treated with 5 mM ATP for 24 h to obtain dead trophoblast cells for phagocytosis assay. THP-1 cells in culture plates were differentiated into macrophages by incubating with 100 ng/ml PMA for 48 h, followed by washing twice with culture media without PMA and a resting period of 24 h, resulting in macrophages with a high phagocytic capacity for phagocytosis assay of apoptotic trophoblast cells. Then, nonadherent macrophages were removed, and apoptotic trophoblast cells were added into the macrophage culture at ratio of 1:3 (macrophages: trophoblasts) in the absence or presence of LXA_4_ (100 nM). After 4-h cultivation, the nonphagocytotic trophoblast cells were washed out, and the macrophages were fixed and stained with CD68 and AT1R antibodies, or subjected to CD68 staining and TUNEL assay. Fluorescence images were processed using Pannoramic MIDI by pannoramic scanner (3D HISTECH, Hungary). The data were analyzed by Caseviewer software.

### Statistical analysis

All statistical analyses were done using the SPSS 19.0 software. The results were expressed as means ± S.E.M of multiple independent experiments. The means of different groups were compared by employing either Student’s *t* test or one-way ANOVA followed by S-N-K post-hoc test. A value of *p* < 0.05 was considered significant.

## Supplementary information

Supplemental figures
