# Supplementary material for: Lipoxin A4 suppresses angiotensin II type 1 receptor autoantibody in preeclampsia via modulating caspase-1
Source: Cell Death Dis. 2020 Jan 30;11(1):78. doi: 10.1038/s41419-020-2281-y (PMC6992755; doi:10.1038/s41419-020-2281-y)
Supplement: Supplementary file 1 — Supplemental figures [file 41419_2020_2281_MOESM1_ESM.pdf]

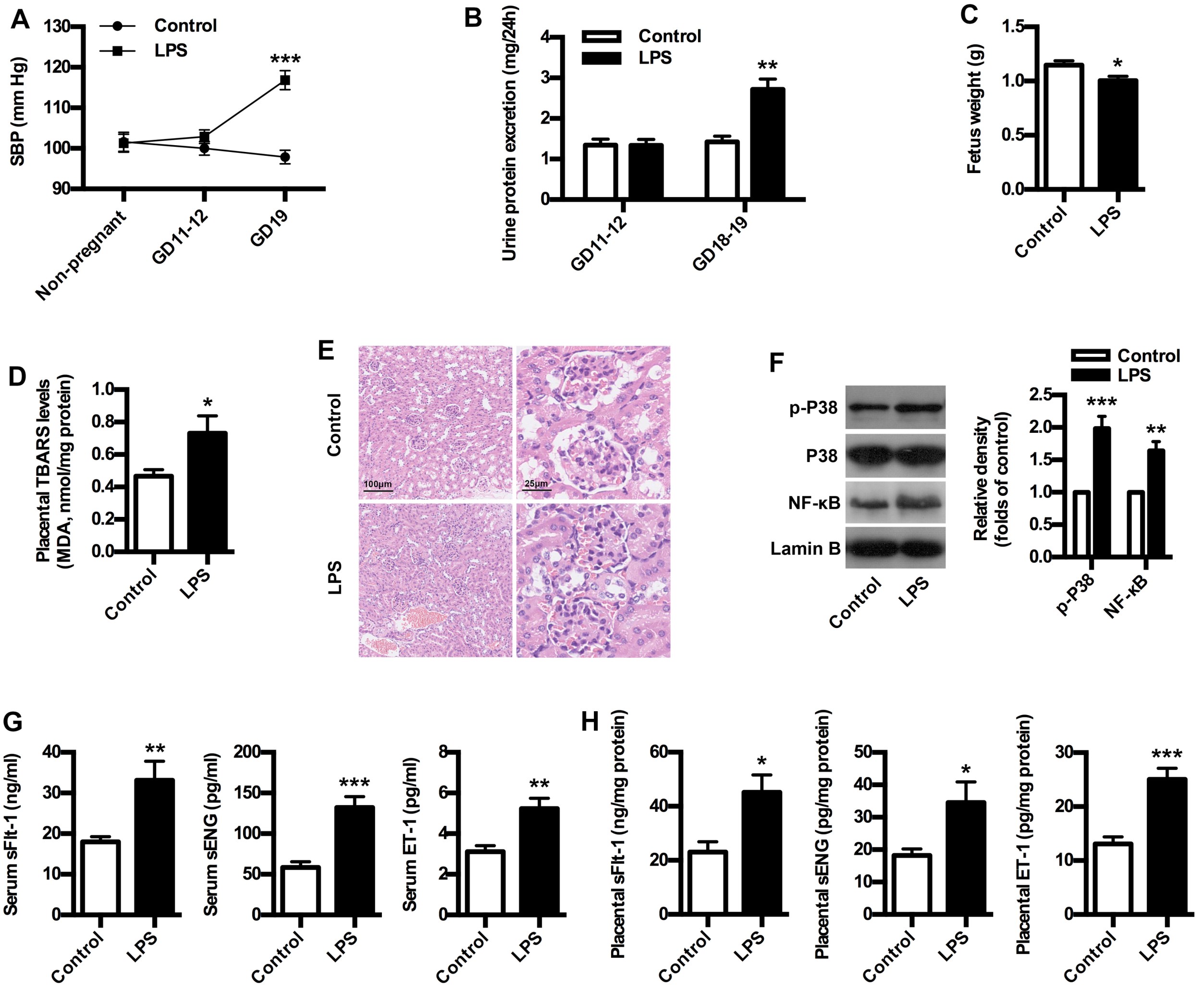

Supplemental Figure 1. Ultra-low-dose LPS induces PE-related manifestations in pregnant mice. Mice were treated with LPS according to *Experimental protocol 1*. SBP, urinary protein excretion and fetal weight are presented in (A)-(C) respectively. (D) Effect of LPS on placental oxidative stress. Degree of placental oxidative stress was determined by measuring the levels of lipid peroxidation using a commercially available TBARS kit**.** (E) Effect of LPS on renal morphological features. Representative H&E staining images of kidney are showed. The bar is 100μm for left two panels, and 25μm for right two panels. (F) Effect of LPS on placental p-P38 and NF-κB expressions. p-P38 and NF-κB were detected by WB. The histogram represents means±SEM of the densitometric scans for protein bands (n=7 mice in each group), normalized

by comparison with P-38 and Lamin B and expressed as a percentage of Control. (G-H) Effect of LPS on levels of sFlt-1, sEng and ET-1 in serum (G) and placenta (H). sFlt-1, sEng and ET-1 were determined by ELISA. Results are expressed as means±SEM (n=7 mice in each group). *p<0.05, **p<0.01 and ***p<0.001 versus control group, two-tailed Student's t-test.

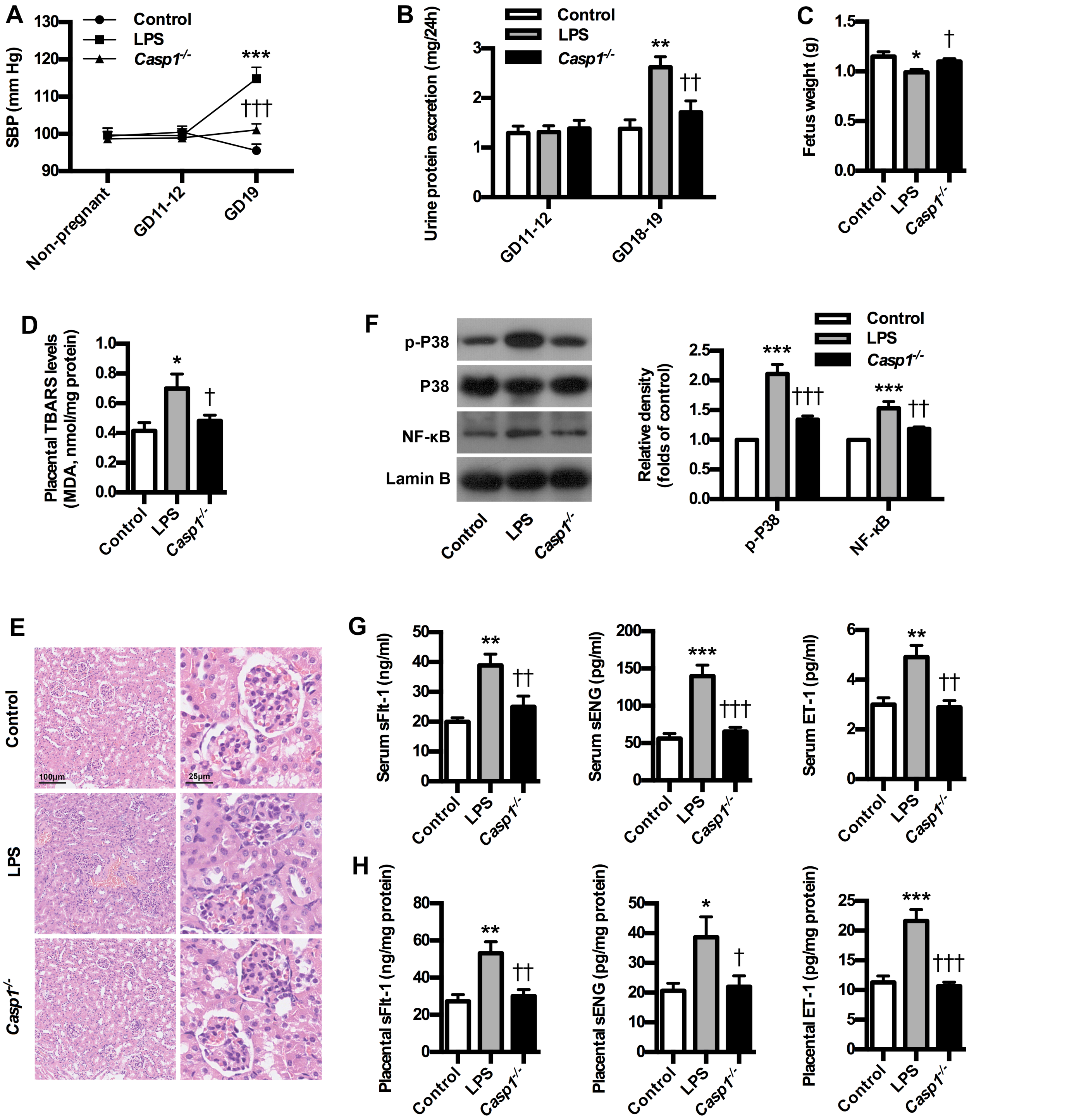

Supplemental Figure 2. Caspase-1 knockout ameliorates PE-related symptoms in pregnant mice. Mice were treated according to *Experimental protocol 2*. SBP, urinary protein excretion and fetal weight are presented in (A)-(C) respectively. (D) Effect of caspase-1 knockout on

placental oxidative stress. Degree of placental oxidative stress was determined by measuring the levels of lipid peroxidation using a commercially available TBARS kit**.** (E) Effect of caspase-1 knockout on renal morphological features. Representative H&E staining images of kidney are showed. The bar is 100μm for left three panels, and 25μm for right three panels. (F) Effect of caspase-1 knockout on placental p-P38 and NF-κB expressions. p-P38 and NF-κB were detected by WB. The histogram represents means±SEM of the densitometric scans for protein bands (n=7 mice in each group), normalized by comparison with P-38 and Lamin B and expressed as a percentage of Control. (G-H) Effect of caspase-1 knockout on levels of sFlt-1, sEng and ET-1 in serum (G) and placenta (H). sFlt-1, sEng and ET-1 were determined by ELISA. Results are expressed as means±SEM (n=7 mice in each group). *P<0.05, **P<0.01 and ***P<0.001 versus control group, †P<0.05, ††P<0.01 and †††P<0.001 versus LPS group, one-way ANOVA with S-N-K posttest.

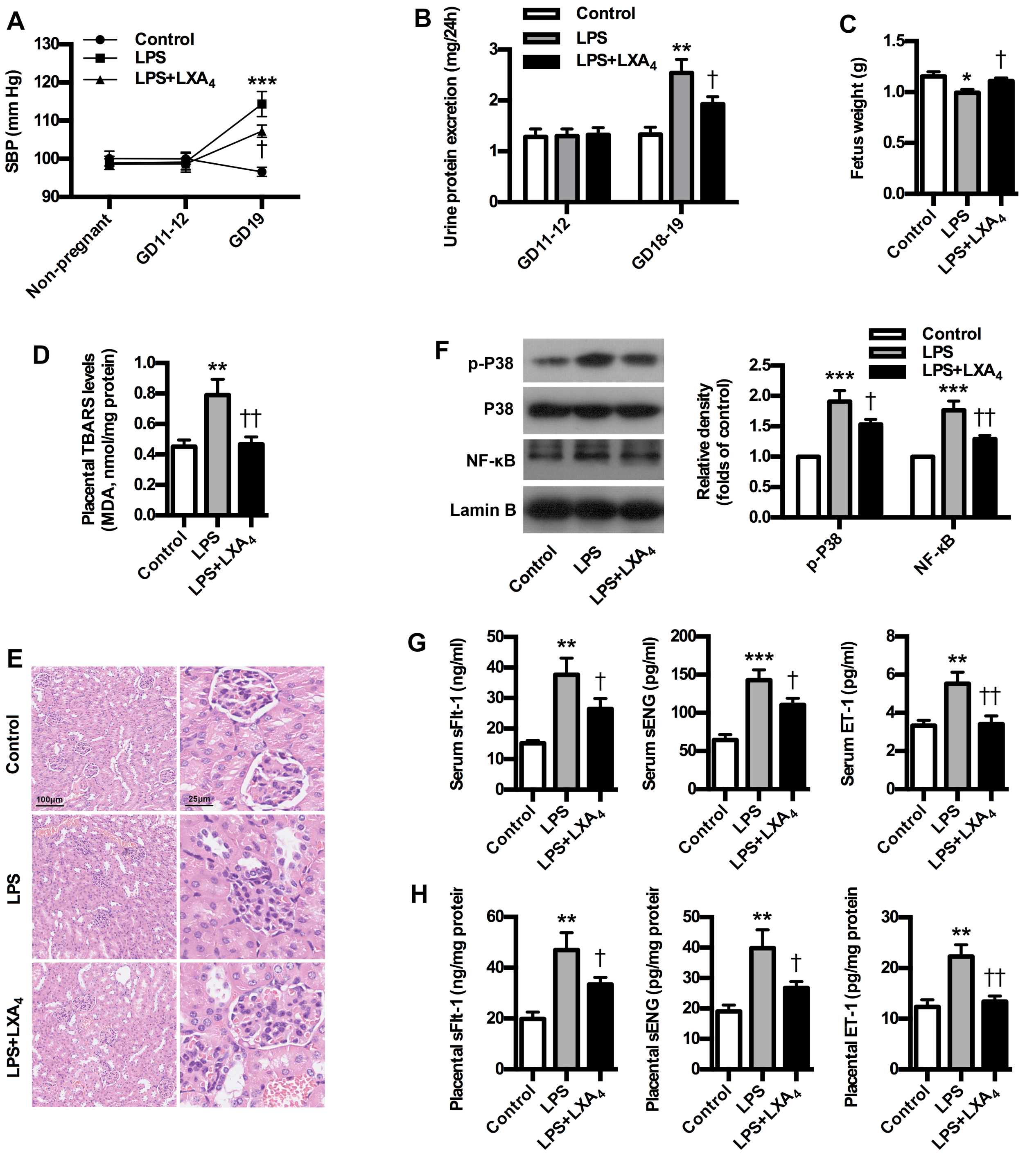

Supplemental Figure 3. LXA_4 improves PE-related symptoms in pregnant mice. Mice were treated according to *Experimental protocol 3*. SBP, urinary protein excretion and fetal weight are presented in (A)-(C) respectively. (D) Effect of LXA_4 on placental oxidative stress. Degree of placental oxidative stress was determined by measuring the levels of lipid peroxidation using a

commercially available TBARS kit. (E) Effect of LXA_4 on renal morphological features. Representative H&E staining images of kidney are showed. The bar is 100μm for left three panels, and 25μm for right three panels. (F) Effect of LXA_4 on placental p-P38 and NF-κB expressions. p-P38 and NF-κB were detected by WB. The histogram represents means±SEM of the densitometric scans for protein bands (n=7 mice in each group), normalized by comparison with P-38 and Lamin B and expressed as a percentage of Control. (G-H) Effect of LXA_4 on levels of sFlt-1, sEng and ET-1 in serum (G) and placenta (H). sFlt-1, sEng and ET-1 were determined by ELISA. Results are expressed as means±SEM (n=7 mice in each group). *$P<0.05$, **$P<0.01$ and ***$P<0.001$ versus control group, $^{\dagger}P<0.05$ and $^{\dagger\dagger}P<0.01$ versus LPS group, one-way ANOVA with S-N-K posttest.
